# Supplementary figures and images for: Fast photostable expansion microscopy using QDots and deconvolution
Source: PLoS One. 2025 Jun 13;20(6):e0325155. doi: 10.1371/journal.pone.0325155 (PMC12165338; doi:10.1371/journal.pone.0325155)

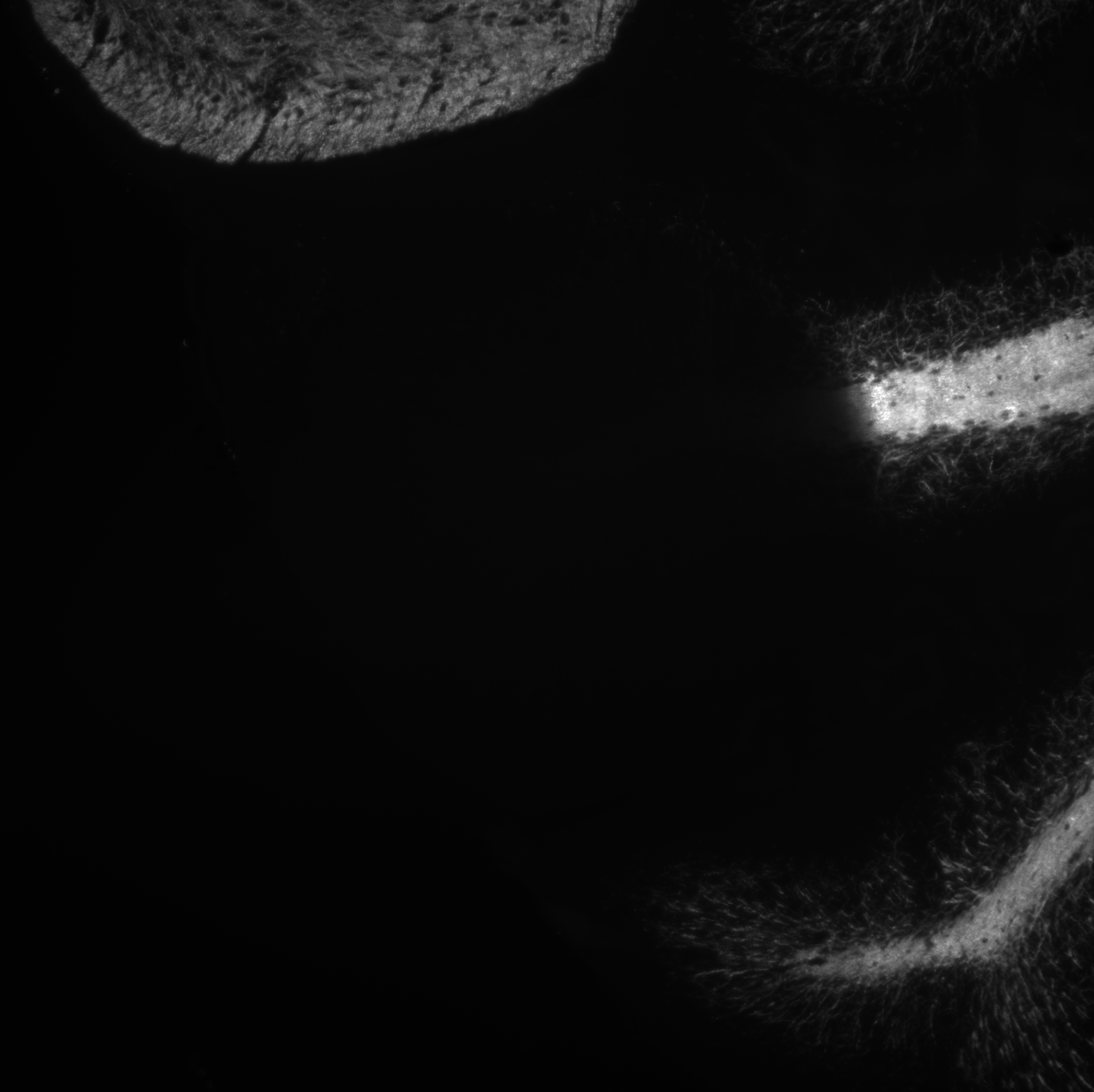

Supplement: S1 Fig — Raw images acquired for Fig 2 showing photobleaching of the Alexa dye. (ZIP) [file pone.0325155.s002.zip › S1 Photostability Images/F2 Alexa after.tif]

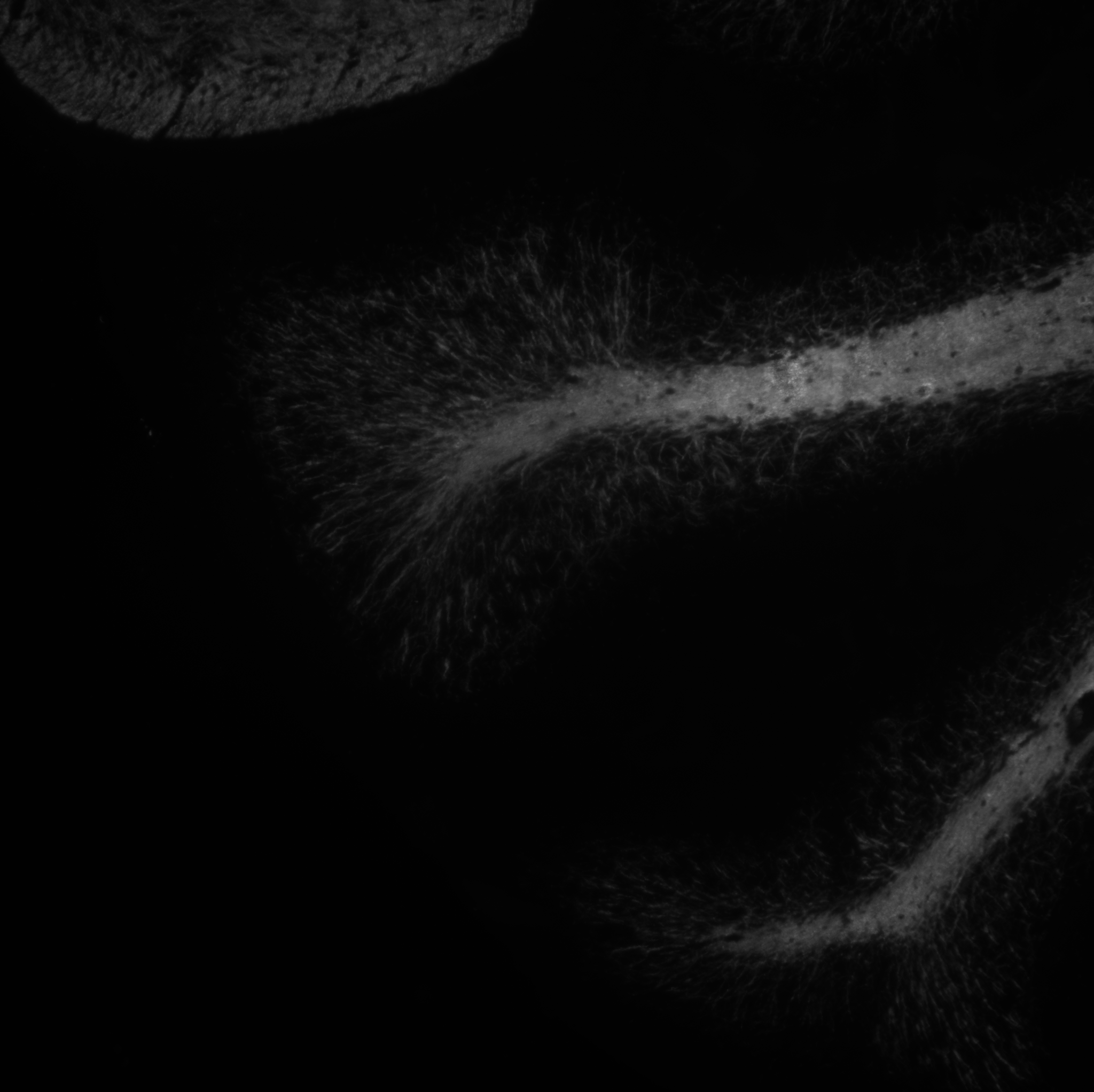

Supplement: S1 Fig — Raw images acquired for Fig 2 showing photobleaching of the Alexa dye. (ZIP) [file pone.0325155.s002.zip › S1 Photostability Images/F2 Alexa before.tif]

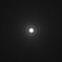

Supplement: S2 Fig — Raw cross-sections of the point spread function used to produce Fig 4. (ZIP) [file pone.0325155.s004.zip › S2 Point Spread Function Data/Figure b.tif]

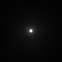

Supplement: S2 Fig — Raw cross-sections of the point spread function used to produce Fig 4. (ZIP) [file pone.0325155.s004.zip › S2 Point Spread Function Data/Figure f.tif]

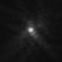

Supplement: S2 Fig — Raw cross-sections of the point spread function used to produce Fig 4. (ZIP) [file pone.0325155.s004.zip › S2 Point Spread Function Data/Figure c.tif]

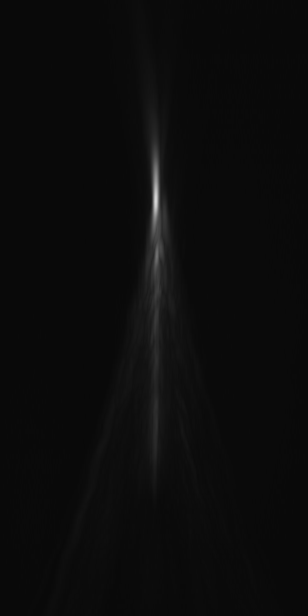

Supplement: S2 Fig — Raw cross-sections of the point spread function used to produce Fig 4. (ZIP) [file pone.0325155.s004.zip › S2 Point Spread Function Data/Figure e.tif]

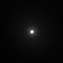

Supplement: S2 Fig — Raw cross-sections of the point spread function used to produce Fig 4. (ZIP) [file pone.0325155.s004.zip › S2 Point Spread Function Data/Figure g.tif]

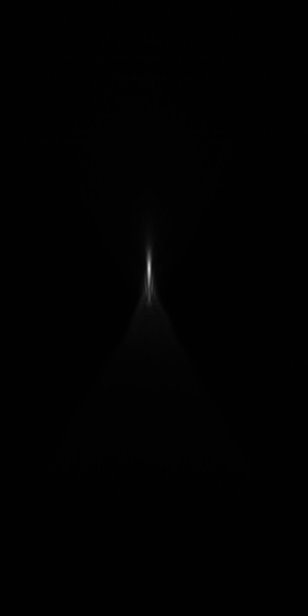

Supplement: S2 Fig — Raw cross-sections of the point spread function used to produce Fig 4. (ZIP) [file pone.0325155.s004.zip › S2 Point Spread Function Data/Figure h.tif]

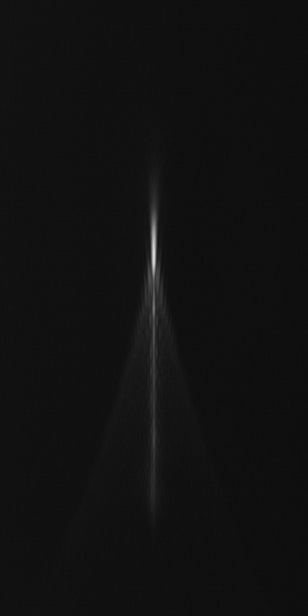

Supplement: S2 Fig — Raw cross-sections of the point spread function used to produce Fig 4. (ZIP) [file pone.0325155.s004.zip › S2 Point Spread Function Data/Figure d.tif]

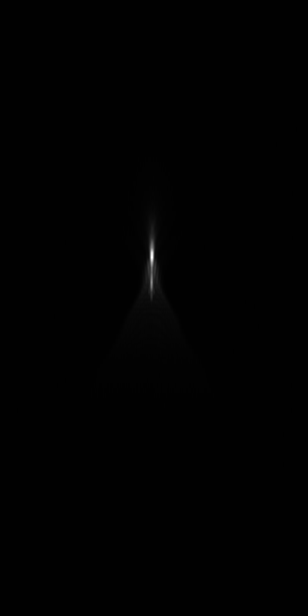

Supplement: S2 Fig — Raw cross-sections of the point spread function used to produce Fig 4. (ZIP) [file pone.0325155.s004.zip › S2 Point Spread Function Data/Figure i.tif]
