# Supplementary material for: Fast photostable expansion microscopy using QDots and deconvolution
Source: PLoS One. 2025 Jun 13;20(6):e0325155. doi: 10.1371/journal.pone.0325155 (PMC12165338; doi:10.1371/journal.pone.0325155)
Supplement: Attachment — Submitted filename: 2025 PLoS QDot revisions.pdf [file pone.0325155.s005.pdf]

Editor

1. Please provide an amended Funding Statement declaring this commercial affiliation, as well as a statement regarding the Role of Funders in your study. If the funding organization did not play a role in the study design, data collection and analysis, decision to publish, or preparation of the manuscript and only provided financial support in the form of authors' salaries and/or research materials, please review your statements relating to the author contributions, and ensure you have specifically and accurately indicated the role(s) that these authors had in your study. You can update author roles in the Author Contributions section of the online submission form.

Please also include the following statement within your amended Funding Statement.

“The funder provided support in the form of salaries for authors [insert relevant initials], but did not have any additional role in the study design, data collection and analysis, decision to publish, or preparation of the manuscript. The specific roles of these authors are articulated in the ‘author contributions’ section.”

If your commercial affiliation did play a role in your study, please state and explain this role within your updated Funding Statement.

We have added this statement into the Cover Letter as requested below (#2).

2. Please also provide an updated Competing Interests Statement declaring this commercial affiliation along with any other relevant declarations relating to employment, consultancy, patents, products in development, or marketed products, etc.

Within your Competing Interests Statement, please confirm that this commercial affiliation does not alter your adherence to all PLOS ONE policies on sharing data and materials by including the following statement: ""This does not alter our adherence to PLOS ONE policies on sharing data and materials.” (as detailed online in our guide for authors http://journals.plos.org/plosone/s/competing-interests) . If this adherence statement is not accurate and there are restrictions on sharing of data and/or materials, please state these. Please note that we cannot proceed with consideration of your article until this information has been declared.

Please include both an updated Funding Statement and Competing Interests Statement in your cover letter. We will change the online submission form on your behalf.

We have moved the Competing Interests Statement and the Funding Statement into the cover letter. We have also verified the metadata in the submission system to ensure that the funding information is correct (see below).

3. Thank you for stating the following in the Acknowledgments Section of your manuscript: [This work was funded in part by:

• (DM) National Institutes of Health / National Heart, Lung, and Blood Institute (NHLBI) #R01HL146745 (https://www.nhlbi.nih.gov/)

• (DM) National Science Foundation CAREER Award #1943455 285

(https://www.nsf.gov/) Sponsors did not play a role in the study design, data collection, analysis, decision to publish, or preparation of the manuscript. DM and JE are both stakeholders in SwiftFront, LLC and WM holds a significant financial interest.]

We note that you have provided funding information that is not currently declared in your Funding Statement. However, funding information should not appear in the Acknowledgments section or other areas of your manuscript. We will only publish funding information present in the Funding Statement section of the online submission form.

We have removed the text from the Acknowledgements section of the paper. We double-checked

the Funding Information section of the submission form and note that the same funding is given (see image).

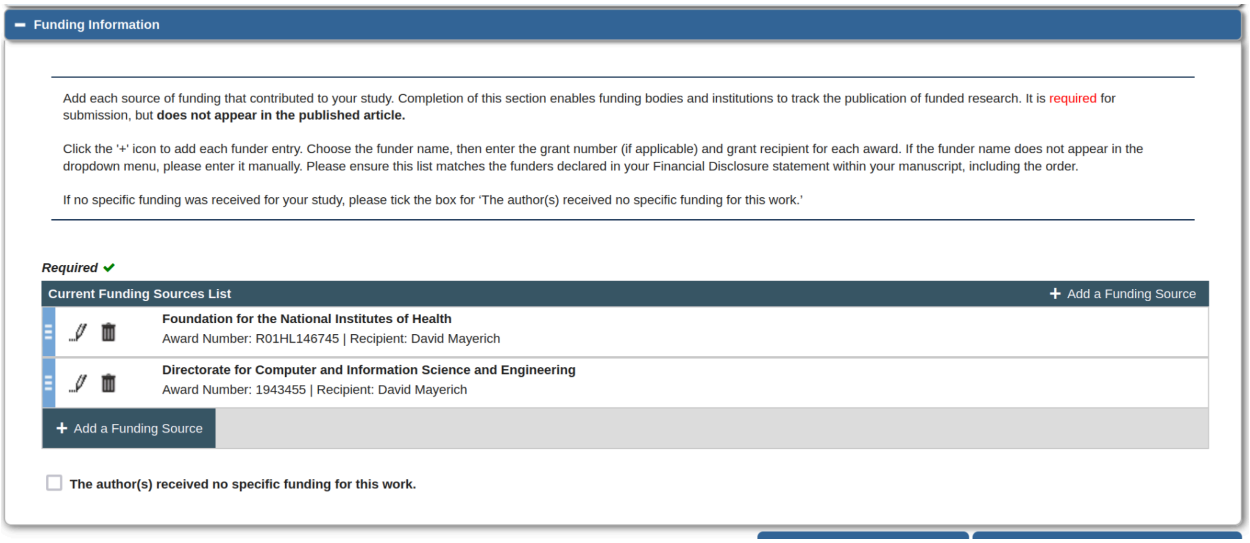
Funding Information

Add each source of funding that contributed to your study. Completion of this section enables funding bodies and institutions to track the publication of funded research. It is required for submission, but **does not appear in the published article.**

Click the '+' icon to add each funder entry. Choose the funder name, then enter the grant number (if applicable) and grant recipient for each award. If the funder name does not appear in the dropdown menu, please enter it manually. Please ensure this list matches the funders declared in your Financial Disclosure statement within your manuscript, including the order.

If no specific funding was received for your study, please tick the box for 'The author(s) received no specific funding for this work.'

Required

Current Funding Sources List	+ Add a Funding Source
**Foundation for the National Institutes of Health**<br>Award Number: R01HL146745 | Recipient: David Mayerich	
**Directorate for Computer and Information Science and Engineering**<br>Award Number: 1943455 | Recipient: David Mayerich	

+ Add a Funding Source

The author(s) received no specific funding for this work.

4. We note that your Data Availability Statement is currently as follows: [All relevant data are within the manuscript and its Supporting Information files.]

Please confirm at this time whether or not your submission contains all raw data required to replicate the results of your study. Authors must share the “minimal data set” for their submission. PLOS defines the minimal data set to consist of the data required to replicate all study findings reported in the article, as well as related metadata and methods (https://journals.plos.org/plosone/s/data-availability#loc-minimal-data-set-definition).

For example, authors should submit the following data:

- The values behind the means, standard deviations and other measures reported;

- The values used to build graphs;

- The points extracted from images for analysis.

Authors do not need to submit their entire data set if only a portion of the data was used in the reported study.

If your submission does not contain these data, please either upload them as Supporting Information files or deposit them to a stable, public repository and provide us with the relevant URLs, DOIs, or accession numbers. For a list of recommended repositories, please see https://journals.plos.org/plosone/s/recommended-repositories.

If there are ethical or legal restrictions on sharing a de-identified data set, please explain them in detail (e.g., data contain potentially sensitive information, data are owned by a third-party organization, etc.) and who has imposed them (e.g., an ethics committee). Please also provide contact information for a data access committee, ethics committee, or other institutional body to which data requests may be sent. If data are owned by a third party, please indicate how others may request data access.

The manuscript contains all of the protocols required to completely duplicate the study. This paper describes a protocol for acquiring three-dimensional images, and does not propose novel data processing or analysis methods. Intermediate steps, such as deconvolution (ex. Figure 5), use commercial software and were performed on three-dimensional images collected using the proposed protocols. While those are too large to upload, they can be readily acquired using the

described protocols.

5. Your abstract cannot contain citations. Please only include citations in the body text of the manuscript, and ensure that they remain in ascending numerical order on first mention.

We have removed all references from the abstract, and any missing ones were placed in appropriate places in the main text.

6. Please review your reference list to ensure that it is complete and correct. If you have cited papers that have been retracted, please include the rationale for doing so in the manuscript text, or remove these references and replace them with relevant current references. Any changes to the reference list should be mentioned in the rebuttal letter that accompanies your revised manuscript. If you need to cite a retracted article, indicate the article's retracted status in the References list and also include a citation and full reference for the retraction notice.

We have checked all references using The Retraction Watch Database to confirm their status, so no changes have been made to the references.

Reviewer 1

The manuscript reports the combination of Expansion microscopy (ExM) with QDots labeling and deconvolution of wide-field images to offer a high-resolution, time-effective imaging approach of biological tissues. The text is well written and easy to follow, therefore I recommend it for publication, after some criticalities have been solved.

1. In general, I find figures to be too many and quite repetitive. I believe that a few figures can be combined together (e.g. Fig2 and 3), resulting in a more fluid reading of the paper.

We have eliminated Figure 1, which is a demonstration of QDot staining on normal tissue and probably unnecessary given their commercial availability.

We have also combined Figure 2 with Figure 3, which makes a pretty seamless transition since it is the same sample.

2. The term "clearing" is not used properly in the text. "Tissue clearing" refers to active processes, whereas what you are referring to as a side effect is more an "increased optical transparency".

We agree with the reviewers and have replaced relevant references to "clearing" with "increased transparency" and "reduced scattering" as appropriate.

3. Line 145: please change the term Alexa with "Alexa-Fluor".

The appropriate name has been updated.

4. I believe it could be of interest for the final goal of the work to include a picture of a brain slice both before and after ExM, taken on graph paper, to confirm the rate of expansion that you measured at the microscale (4.1x).

The practical problem here is our ability to precisely measure the expansion using macro-scale features, such as graph paper. This figure has been shown in prior publications to demonstrate ExM, and therefore may be redundant here. In order to precisely measure the expansion factor, compare microscopic features to achieve the necessary precision. In our case, that is done using images like those shown in Figure 9.

5. In paragraph 3.4, could you please provide information on the laser intensity used, and if the measurements were performed at the same intensity?

These images are collected using an incoherent (LED) source that is unchanged during the

duration of the time series. It is difficult to replicate the exact power from incoherent sources, because they are affected by a diverse range of optical properties. However, they can be kept constant throughout an experiment, as has been done here.

6. Figure 4C is not clear to me. Why are the structures at the border brighter than in figure 4B? if this is due to modified contrasts, it is more correct to use the same settings in the two images. Moreover, the reference to panel C is missing in the caption.

This intensity change was an artifact introduced by the microscope software auto-adjusting to maintain a constant global image intensity. We've updated the images with the appropriate constant intensity. We have also added "(c)" to the appropriate place in the caption.

7. Finally, the conclusion section does not add anything to the paper, except for summarizing the results. Please, discuss your findings and present further improvements that can be of interest. For example, in lines 239-243, you discuss how changing the refractive index can improve the outcome. It is also possible to match the refractive index of the expanded samples, and use a RI tunable lens for the imaging for better results.

The Conclusion has been completely re-written to incorporate challenges such as (1) the potential for QDot materials being incompatible with DI water and (2) the current reliance on secondary detection, which can be time consuming for multiplex labeling.

Reviewer #2: The manuscript presents a novel approach to combining quantum dots (QDots) with expansion microscopy (ExM) to enhance imaging speed and photostability for super-resolution imaging. The study demonstrates the utility of QDots for multiplexed labeling and widefield imaging and validates their superiority over traditional fluorophores in terms of photostability and signal retention. The methodology and results are well-detailed and provide a significant advancement for researchers in microscopy and imaging sciences.

I really appreciate the novelty of the study and the comprehensive methodology. The use of QDots in ExM is a clear innovation that addresses challenges such as photobleaching and acquisition speed. The application of deconvolution further enhances the utility of the protocol.

Minor comments:

The introduction is well-written but assumes some familiarity with ExM. Adding a brief explanation of why ExM inherently reduces fluorescence signal and how QDots counteract this would enhance accessibility for readers unfamiliar with the technique.

We added the following explanation in the second paragraph of the Introduction: The decrease in fluorophore density, combined with digestion-induced quenching, decreases signal-to-noise ratio (SNR) and lengthens acquisition time. Widefield imaging can compensate by using a broadband incoherent light source. However, this increases illumination incident on the sample, causing photobleaching while reducing z-axis resolution. We demonstrate that these limitations can be improved by combining quantum dots (QDots) with widefield deconvolution, while taking advantage of the reduced scattering that occurs with ExM. This combined approach makes widefield imaging practical, since QDots minimize photobleaching, while deconvolution improves axial resolution.

I understand that the manuscript offers clear qualitative comparisons, such as photostability and resolution improvements. While a more quantitative evaluation of imaging resolution enhancement post-deconvolution (e.g., statistical analysis of FWHM across multiple samples) is not strictly necessary, it

would significantly strengthen the claims.

Cross-sectional plots were added to (current) Figures 4 and 5 showing a one-dimensional function of the PSF and spatial features in the images.

The manuscript concludes with a strong emphasis on the utility of QDots for ExM but could benefit from a broader discussion of potential limitations (e.g., QDot aggregation or cost) and possible solutions.

We re-wrote the conclusion including key issues that can arise from QDots (see above).
