# Supplementary material for: Fast photostable expansion microscopy using QDots and deconvolution
Source: PLoS One. 2025 Jun 13;20(6):e0325155. doi: 10.1371/journal.pone.0325155 (PMC12165338; doi:10.1371/journal.pone.0325155)
Supplement: Attachment — Submitted filename: PLOS QDots + ExM Response 2.pdf [file pone.0325155.s006.pdf]

Reviewer's Responses to Questions

Comments to the Author

Reviewer #1: I recommend this revised version of the paper for publication as the authors have adequately answered and revised the weaknesses found in the previous round of revision.

Reviewer #2: The manuscript presents a method that integrates quantum dots (QDots), widefield imaging, and deconvolution to improve photostability and acquisition speed in expansion microscopy (ExM). The study is relevant, as traditional ExM techniques suffer from signal dilution and photobleaching, which limit their practical use for large-scale, high-resolution imaging. The authors propose that combining QDots with widefield imaging and deconvolution overcomes these limitations, offering a more efficient approach.

Overall, the manuscript is well-structured and clearly written, with appropriate methodology and data presentation. However, some areas require further clarification and improvement with minor revision.

We thank the reviewers for their time and insightful work on this review.

o Deconvolution Parameters: While the authors apply Huygens software for deconvolution, the exact PSF modeling parameters are missing.

We use Huygens Professional (20.10) classical maximum likelihood estimation (CMLE) for blind deconvolution. Exact modeling parameters have now been included in the manuscript (lines 224-229):

“Deconvolution was performed using Huygens (Scientific Volume Imaging) version 20.10 with classic maximum likelihood estimation (CMLE). Regularization parameters were tuned by adjusting the SNR between 30-40, and the background level was evaluated using the automated approach in the Deconvolution Wizard, with 94 iterations and a quality threshold of 0.02.”

o Photostability Comparisons: The time-lapse studies are valuable, but it would be useful to include a quantitative photostability index for QDots vs. Alexa dyes across multiple imaging sessions.

The QDots used in this study are a proprietary label developed by Invitrogen for histological applications. Unfortunately, we lack the equipment and expertise to quantify the quantum efficiency of these particles. Our primary goal with the time-lapse experiments was to demonstrate that QDots behave as expected in the aqueous environment required for expansion microscopy. However, thorough studies comparing these proprietary QDots with Alexa Fluor fluorophores have been reported:

Nikiforov, Theo T., and Joseph M. Beechem. "Development of homogeneous binding assays based on fluorescence resonance energy transfer between quantum dots and Alexa Fluor fluorophores." Analytical Biochemistry 357.1 (2006): 68-76.

To clarify this point, we have added lines 182-185 to our manuscript and cited this reference [29].

o The manuscript reports improvement in signal-to-noise ratio (SNR), but no statistical tests are provided to support this claim.

We have included the signal-to-noise ratio across all images in Figure 3. The SNR is expressed in decibels, and the noise was calculated by averaging the noise in an empty region of the images. We also noticed that the contrast scaling in the original figure was confusing. We set a constant contrast range for all images in the sequence, so that it is clear that the change in SNR is due to a reduced signal from photobleaching. We have adjusted the legend of Figure 3 accordingly.

o A side-by-side quantitative comparison of SNR before and after deconvolution (e.g., mean intensity,

contrast-to-noise ratio) should be included.

The SNR after deconvolution would be misleading because this value is used as the criteria to end the iterative deconvolution process. Essentially, the SNR of the deconvolved image is an input parameter into the deconvolution algorithm. This SNR was provided above and added to the paper.

o In Fig. 7 (pre- and post-deconvolution images), an objective numerical comparison of resolution enhancement (e.g., FWHM of PSFs before and after processing) should be provided.

Both of the images in Figure 7 are post-deconvolution, and show the subjective improvement in visible astrocyte structures with and without expansion microscopy. It is difficult to compare two deconvolved results objectively without an underlying ground-truth since both images are reconstructed algorithmically. However, we attempted to make an objective comparison between pre- and post-deconvolution images in Figure 5 by showing plots of the numerical intensity profiles. We have adjusted the legend of Figure 5 to clarify this.

Recommendation: the study presents a novel application of QDots in ExM, but methodological gaps, data quantification, and statistical analysis should be improved before publication. Addressing the quantitative comparisons, SNR/statistical validation, and deconvolution parameters would strengthen the manuscript.
